# The Genome Sequence of the Highly Acetic Acid-Tolerant *Zygosaccharomyces bailii-*Derived Interspecies Hybrid Strain ISA1307, Isolated From a Sparkling Wine Plant

**DOI:** 10.1093/dnares/dst058

**Published:** 2014-01-21

**Authors:** Nuno P. Mira, Martin Münsterkötter, Filipa Dias-Valada, Júlia Santos, Margarida Palma, Filipa C. Roque, Joana F. Guerreiro, Fernando Rodrigues, Maria João Sousa, Cecília Leão, Ulrich Güldener, Isabel Sá-Correia

**Affiliations:** 1 IBB-Institute for Biotechnology and Bioengineering, Center for Biological and Chemical Engineering, Instituto Superior Técnico, Department of Bioengineering, Universidade de Lisboa, Avenida Rovisco Pais, Lisbon 1049-001, Portugal; 2 Institute of Bioinformatics and Systems Biology, Helmholtz Zentrum München, German Research Center for Environmental Health (GmbH), Ingolstädter Landstrasse 1, Neuherberg D-85764, Germany; 3 Life and Health Sciences Research Institute (ICVS), School of Health Sciences, University of Minho, Braga 4710-057, Portugal; 4 ICVS/3B's-PT Government Associate Laboratory, Life and Health Sciences Research Institute (ICVS), School of Health Sciences, University of Minho, Braga 4710-057, Portugal; 5 Centre of Molecular and Environmental Biology (CBMA)/Department of Biology, University of Minho, Braga 4710-057, Portugal

**Keywords:** *Zygosaccharomyces bailii*, hybrid yeast strains, weak acid food preservatives tolerance, wine yeast strains, genome sequencing and annotation

## Abstract

In this work, it is described the sequencing and annotation of the genome of the yeast strain ISA1307, isolated from a sparkling wine continuous production plant. This strain, formerly considered of the *Zygosaccharomyces bailii* species, has been used to study *Z. bailii* physiology, in particular, its extreme tolerance to acetic acid stress at low pH. The analysis of the genome sequence described in this work indicates that strain ISA1307 is an interspecies hybrid between *Z. bailii* and a closely related species. The genome sequence of ISA1307 is distributed through 154 scaffolds and has a size of around 21.2 Mb, corresponding to 96% of the genome size estimated by flow cytometry. Annotation of ISA1307 genome includes 4385 duplicated genes (∼90% of the total number of predicted genes) and 1155 predicted single-copy genes. The functional categories including a higher number of genes are ‘Metabolism and generation of energy’, ‘Protein folding, modification and targeting’ and ‘Biogenesis of cellular components’. The knowledge of the genome sequence of the ISA1307 strain is expected to contribute to accelerate systems-level understanding of stress resistance mechanisms in *Z. bailii* and to inspire and guide novel biotechnological applications of this yeast species/strain in fermentation processes, given its high resilience to acidic stress. The availability of the ISA1307 genome sequence also paves the way to a better understanding of the genetic mechanisms underlying the generation and selection of more robust hybrid yeast strains in the stressful environment of wine fermentations.

## 1. Introduction

Among food spoilage yeasts, those belonging to the genus *Zygosaccharomyces* are considered the most problematic to the food and beverage industries, with the *Z. bailli* species representing the most significant spoilage yeast within the genus, specially in acidic food products.^1,2^ Regardless of the progress achieved in product formulation and in the control and development of improved sanitation technologies, *Z. bailii* still is a major challenging threat of spoilage in mayonnaise, salad dressings, sauces, pickled or brined vegetables, fruit concentrates and various non-carbonated fruit drinks as well as other acidified foods.^1,2^ *Zygosaccharomyces bailii* is also a significant spoiler of wines.^3^ The success of *Z. bailii* as a spoilage yeast results from a number of physiological traits of the species, in particular, its remarkable resilience against weak acids used as food preservatives such as acetic, benzoic, propionic, sorbic acids and sulphur dioxide, being able to proliferate in the presence of concentrations which are frequently above the permitted values by some food legislations.^1,2,4^ *Zygosaccharomyces bailii* is also able to tolerate high concentrations of ethanol and other sanitizers and to grow in a wide range of pH (2.0–7.0) and water activities (0.80–0.99).^1,2^ *Zygosaccharomyces bailii* is known to vigorously ferment hexoses and, like other members of the *Zygosaccharomyces* genus, *Z. bailii* exhibits a frutophilic behaviour metabolizing fructose at a higher rate than glucose when the two carbon sources are present in the growth medium.^2,5,6^ Moreover, *Z. bailii* is able to cause spoilage from an extremely low inoculum, to tolerate moderate osmotic pressure and to grow at high growth rates under oxygen-restrictive conditions.^2,7^ Food products that are preserved at low pH, low water activities or low oxygen concentrations, and that contain adequate amounts of fermentable sugars, are therefore at a particular risk of spoilage by this yeast, causing significant economic losses for the industries that produce and commercialize these products. *Zygosaccharomyces bailii* is also frequently isolated in wine fermentations and although this is generally considered detrimental, potential beneficial effects have also been proposed.^8,9^ This yeast species is a potential new host for biotechnological processes.^10,11^ In particular, it is an attractive candidate to allow fermentation processes to be performed under otherwise restrictive conditions, or to be used in heterologous protein and metabolite production due to its high resilience to a number of environmental stresses, high specific growth rate and high biomass yield.^10,11^ The use of *Z. bailii* was already found to be successful for the production of lactic acid, l-ascorbic acid (vitamin C) and vitamin B12.^11,12^

Differently from *Saccharomyces cerevisiae*,^13^ the exploitation of Omic strategies in *Zygosaccharomyces* yeasts has been severely limited by the absence of available genome sequences for species of this genus. The genome of *Z. rouxii* CBS732, completed in 2009,^14^ was the first genome sequence of this genus being disclosed and only very recently the genome sequence of the *Z. bailii* type strain CLIB213^T^ (=ATCC58445), was released.^15^ Therefore, until today, most of the studies dedicated to *Z. bailii* only explored gene-by-gene approaches.^7,16–20^ A quantitative proteomic analysis, based on quantitative two-dimensional gel electrophoresis (2-DE), was however recently performed to elucidate the mechanisms underlying the adaptive response and intrinsic high tolerance of *Z. bailii* cells to sub-lethal concentrations of acetic acid.^21^ A coordinate increase in the content of proteins involved in carbohydrate metabolism and energy generation as well as in general and oxidative stress response was registered.^21^ Results reinforced a previously established concept that glucose and acetic acid are co-consumed in *Z. bailii*, with acetate being channelled into the tricarboxylic acid cycle.^18,21,22^ When acetic acid is the sole carbon source, results suggest the activation of gluconeogenic and pentose phosphate pathways, based on the increased content of several proteins of these pathways after glucose exhaustion.^21^ The lack of a genome sequence for *Z. bailii* limited this expression proteomic analysis, given that only 40% of the differently expressed proteins could be identified by peptide mass fingerprinting.^21^ The development of molecular biology tools for *Z. bailii*, such as the isolation of stable auxotrophic mutants and release of a set of vectors allowing ectopic gene expression, is also relatively recent.^10^

In this article, we describe the sequencing and annotation of strain ISA1307, isolated from a continuous sparkling wine production plant.^23^ Here, we also provide evidences supporting the notion that this strain, formerly considered of the *Z. bailii* species, is an interspecies hybrid between *Z. bailii* and another closely related yeast species. The phylogenetic relationships of a large cohort of isolates first classified as *Z. bailii* were recently re-examined, and significant differences in their rRNA gene sequences and genome fingerprinting patterns were found, leading to the distribution of these isolates into three species: *Z. bailii*, *Z. parabailii* and *Z. pseudobailii*.^24^ Despite the differences registered at the molecular level, the *Z. bailii* species could not be distinguished from the other two novel species using physiological tests.^24^ The occurrence in wines of natural hybrid strains generated by hybridization of different *Saccharomyces* species is widely described in the literature,^25,26^ the lagger brewing yeast *Saccharomyces pastorianus* being the most paradigmatic example.^27^ The occurrence of hybrid strains within the *Zygosaccharomyces* genus involving, at least, the *Z. rouxii*, *Z. pseudorouxii* and *Z. mellis* species, was also reported.^28–30^ The ISA1307 strain focused on our work has been used in several studies conducted to examine different aspects of *Z. bailii* physiology, in particular, its extreme tolerance to acetic acid (minimum inhibitory concentration value for acetic acid in the range of 270–420 mM compared with 80 mM for *S. cerevisiae*^21,31^ and our unpublished results), metabolism of fructose and glucose^5,32^ and growth under oxygen-restrictive conditions.^7^ A genomic library from strain ISA1307 was constructed^33^ and successfully used for functional analysis of several relevant genes.^5,19^ Considering that formation of hybrids in the stressful environment of wine fermentations has been associated with improved strain robustness strains,^25^ it is expected that the sequencing and annotation of ISA1307 genome reported in this work may be used to inspire and guide novel biotechnological applications of this strain and *Z. bailii* species under otherwise restrictive process conditions. The availability of the ISA1307 genome sequence will also open the door to a better understanding of the genetic mechanisms underlying the generation of hybrid strains in the stressful environment of wine fermentations.

## 2. Materials and methods

### 2.1. Strains and growth medium

The prototrophic yeast isolates ISA1307,^23^ *Z. bailii* ATCC58445^T^ (=CLIB213^T^) and the laboratory strains acquired from the Euroscarf collection *S. cerevisiae* BY4741 (genotype MATα; *his3*Δ 1; *leu2*Δ 0; *lys2*Δ 0; *ura3*Δ 0) and *S. cerevisiae* BY4743 (genotype *MAT*a/α *his3*Δ*1/his3*Δ*1 leu2*Δ*0/leu2*Δ*0 LYS2/lys2*Δ*0 met15*Δ*0/MET15 ura3*Δ*0/ura3*Δ*0*) were used in this study. Strains were maintained and cultivated in rich YPD growth medium which contains, per liter, 2% glucose (Merck), 2% yeast extract (Difco) and 2% peptone (Difco).

### 2.2. Quantification of ISA1307 and S. cerevisae total genomic DNA by flow cytometry

**Figure 1.**
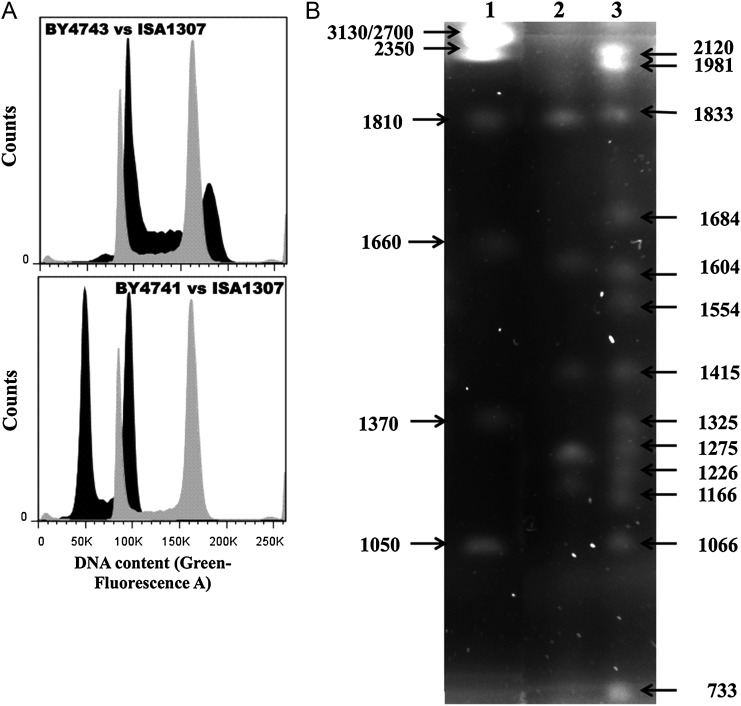
Estimation of genome size and karyotyping of the ISA1307 strain. (A) Representative cell cycle analysis histogram of *S. cerevisiae* BY4741 or BY4743 (in black) and ISA1307 (in grey). ISA1307 and *S. cerevisiae* cells were cultivated in YPD growth medium until stationary phase and then labelled with SYBR Green I to stain genomic DNA. Mean fluorescent intensities (MFI) of G_0_/G_1_ peaks of the cell cycle histogram were estimated by flow cytometry. The MFI values obtained for the two *S. cerevisiae* strains were used to build a calibration curve that was used to calculate the size of the genome of the ISA1307 strain (12.16 Mb for the size of the genome of the haploid strain *S. cerevisiae* BY4741). (B) Karyotype of the reference strain *Z. bailii* ATCC58445 (lane 2) and of the ISA1307 strain (lane 3). Total genomic DNA of both yeast species cultivated in YPD growth medium until stationary phase was separated by PFGE. The size of ISA1307 high-molecular-weight chromosomes was estimated based on the high-molecular-weight standard [*Hansenula wingei* (Bio-Rad)—lane 1], while the size of low-molecular-weight chromosomes was estimated using *S. cerevisiae* chromosomes' size (not shown).

Quantification of total genomic DNA from *S. cerevisiae* (strains BY4741 and BY4743) and of the hybrid strain ISA1307 was performed using a SYBR Green I-based staining protocol, as described before.^34^ Briefly, cells batch cultured in YPD growth medium, at 26°C, until mid-exponential phase (OD_600 nm_ of 1.0 ± 0.01; 10^6^ cells for each species), were harvested by centrifugation, washed with H_2_O and fixed overnight in 0.5 ml of 70% ethanol (vol/vol). Fixed cells were collected by centrifugation, washed with 50 mM of sodium citrate buffer (pH 7.5) and re-suspended in 750 μL of this same buffer supplemented with 1 mg of RNAse A. After 1 h of incubation at 50°C, 1 mg of proteinase K was added to the cell suspension and the mixture was left at 50°C for another hour. Cells were subsequently stained using 20 μL of SYBR Green I working solution (corresponding to a 500-fold dilution of the commercial solution). Samples were sonicated at low power and analysed in an Epics^®^ XL™ (Beckman Coulter) flow cytometer equipped with an argon ion laser emitting a 488-nm beam at 15 mW. The green fluorescence was collected through a 488-nm blocking filter, a 550-nm/long-pass dichroic and a 525-nm/bandpass. Thirty thousand cells per sample were analysed to obtain the cell cycle profiles shown in Fig. 1. The mean fluorescent intensities obtained for *S. cerevisiae* BY4741 and BY4743 were used to build a calibration curve from which it was estimated the size of the genome of the ISA1307 strain.

### 2.3. Karyotyping of the ISA1307 strain

Intact DNA for pulsed field gel electrophoresis (PFGE) was prepared in plugs as previously described.^35^ ISA1307 and *Z. bailii* ATCC58445 cells, cultivated overnight at 26°C in YPD growth medium, were harvested by centrifugation, washed twice with 0.05 M EDTA, pH 8.0 and resuspended at a concentration of 1.2 × 10^9^ cells/ml in 0.05 M EDTA containing 3 mg/ml of Zymolyase 100T for digestion. Plugs were formed by mixing the suspension of cells with the same volume of low melting agarose 2% (SeaPlaque; Cambex Bio Science, Rockland, ME, USA) at 40°C. Plugs were then incubated overnight in 0.45 mM EDTA, pH 8.0 and 7.5% (vol/vol) 2-mercaptoethanol at 37°C. After this incubation step, plugs were washed three times in Tris/EDTA buffer (10 mM Tris, pH 8.0 and 1 mM EDTA, pH 8.0) and incubated overnight in 0.5 M EDTA, 10 mM Tris, pH 8.0, 1 mg/mL of proteinase K (Sigma-Aldrich) and 1% sodium-*N*-lauryl sarcosinate at 50°C. After washing five times, during 30 min each, with TE, pH 8.0, at room temperature, samples were stored at 4°C. PFGE was performed in a CHEF-DRII Chiller System (Bio-Rad, Hercules, CA, USA). PFGE gels were run in 0.5% Tris borate–EDTA buffer at 12°C with an angle of 120° with a voltage of 3 V/cm and switch times of 300 s for 120 h.

### 2.4. Genome sequencing, assembly and annotation

The genome of the ISA1307 hybrid strain was sequenced at CD Genomics (New York, USA) using a whole-genome shotgun approach that explored paired-end Illumina sequencing. Details on the methods used for genome sequencing, assembly and subsequent annotation are described in Supplementary Material.

## 3. Results and discussion

### 3.1. The ISA1307 strain is an interspecies hybrid between *Z*. *bailii* and a closely related species

Following the analysis of the ISA1307 strain genome sequence described below and given that yeast isolates formerly identified as *Z. bailii* were recently reclassified in the *Z. bailii*, *Z. parabailii* and *Z. pseudobailii* species,^24^ we have examined the taxonomic classification of this strain. The sequences of the house-keeping genes *RPB1*, *RPB2*, *EF1-α* and β-tubulin were compared. These gene sequences were proposed as sequences with a very high capacity to discriminate *Z. bailii*, *Z. parabailii* and *Z. pseudobailii* species.^24^ Only one copy of the *RPB1* gene was found in the ISA1307 genome, this being identical to the corresponding orthologue annotated in the genome of several *Z. bailii* strains (Supplementary Material). The β-tubulin, *RPB2* and *EF1*-*α* genes are duplicated in the genome of the ISA1307 strain, with one allele being almost identical (>99% identity at the nucleotide level) to the corresponding orthologue found in *Z. bailii* strains and the other allele being identical to the orthologues found in *Z. parabailii* strains^24^ (Supplementary Material). The analysis of the genome sequence revealed that this allelic divergence is registered in ∼90% of the genes found to be duplicated in the ISA1307 genome (see below). Altogether, these results strongly suggest that the ISA1307 strain is an interspecies hybrid between *Z. bailii* and a closely related species. The results obtained for sequences of β-tubulin, *EF1-α* and *RPB2* genes appear to suggest that *Z. parabailii* could be the other parental species. A closer inspection to the sequences of these genes deposited for the different strains classified as *Z. parababilii* by Suh *et al.*^24^ showed the existence of multiple ambiguous positions, which suggests that these sequences already have been obtained by amplification of divergent alleles. Thus, we hypothesize that the strains previously classified as *Z. parabailii* could be hybrid strains. This hypothesis is in line with the reported inability of *Z. parabailii* ATCC56075 (=NCYC128) to undergo meiotic sporulation,^36^ a phenotypic trait common in hybrid strains^25,37^ and also described for ISA1307.^17^

### 3.2. Karyotyping and estimation of total DNA content of the ISA1307 strain

To estimate the size of ISA1307 genome, exponential cells were fixed and DNA was quantified by flow cytometry using the fluorescent probe SYBR Green I.^17^ Cell cycle analysis revealed that the intensity of the G_0_/G_1_ peak exhibited by ISA1307 cells is 1.7-fold higher and 1.1-fold lower than the values registered for the reference strains *S. cerevisiae* BY4741 (haploid) and BY4743 (diploid), respectively (Fig. 1A). Considering that *S. cerevisiae* BY4741 has a size of 12.16 Mb (www.yeastgenome.org), the estimated size of ISA1307 total DNA is ∼22.0 Mb (Table 1). To complement this analysis, PFGE was used to separate ISA1307 genomic DNA. Under the experimental conditions used, 13 chromosomal bands were observed, with sizes ranging from 733 to 2120 Mb (Fig. 1B). PFGE profiling of the type strain *Z. bailii* ATCC58445 (=CLIB213^T^) was also performed, and five chromosomal bands were observed (Fig. 1B). This result is in line with a previous publication, suggesting that the ISA1307 strain has at least three more chromosomes than the *Z. bailii* type strain.^17^ The sum of the PFGE bands is ∼19 Mb, differing by 3 Mb of the total amount of DNA that was estimated by flow cytometry, a gap that can result from co-migration of chromosomal bands in the PFGE gel. In fact, it is possible that the first two bands (2120 and 1981 kb) and eventually the last band (730 kb) are duplicated, based on their higher intensity, compared with the other bands observed in the gel (Fig. 1).

**Table 1.** Genome assembly statistics of the *Z. bailii-*derived interspecies hybrid strain ISA1307

Total reads	120 000 000
No. of scaffolds	154
Coverage	×600
N50 (bp)	232 974
Maximum contig length (bp)	806 952
Minimum contig length (bp)	2160
Average contig length (bp)	137 280
Assembly size (bp)	21 141 152

The most significant parameters associated with assembly of the reads that were obtained after sequencing of the ISA1307 genome are indicated.

### 3.3. Assembly of ISA1307 genome

Two rounds of paired-end Illumina sequencing (inserts with ∼350 bp, 100 base reads) were carried out to obtain the sequence of ISA1307 genome. Around 120 Gb of readings were acquired yielding a genome coverage of 600 fold. The *de novo* assembly of the reads was carried out using SOAPde novo assembler^38^ resulting in 190 scaffolds. After the assembly process, the sum of the scaffolds size obtained (21.1 Mb) was well above the size expected for a haploid genome (which would be ∼11 Mb), indicating that the duplicated sequences from the homeologous chromosomes (homologous chromosomes acquired from two different species) of the ISA1307 strain were not aligned in a unique consensus sequence. The same had also been obtained during genome sequencing of other interspecies hybrid strains, such as *S. pastorianus* or *Pichia sorbitophila*,^27,39^ this being attributed to the different origin of the homeologous chromosomes that compose the genome of hybrid strains. To reconstruct the genome sequence of the ISA1307 strain, we have used a similar approach to the one used to assemble the genome of other hybrid yeast strains.^27,39,40^ Briefly, 190 scaffolds with homologous genes were detected (using an all-against-all BLASTP analysis) and then sequentially ordered based on the search of syntenic blocks with the genomes of *Z. rouxii* CBS732 and *S. cerevisiae* S288c. These yeast species were selected for this analysis, since they are phylogenetically close to *Z. bailii* and their genomes are well annotated and available in public databases (Genolévures database^41^ and *Saccharomyces* Genome database^42^ or CYGD,^43^ respectively). The junction points between scaffolds predicted to be contiguous by our synteny-based *in silico* analysis were tested by PCR to confirm correct scaffold positioning, and the existing gaps were closed by sequencing the amplification product. A summary of the genome assembly statistics is summarized in Table 1. The final reconstructed genomic sequence of the ISA1307 strain is distributed over 154 scaffolds with sizes ranging from 2160 to 806 952 bp. The sum of all scaffolds size is 21 141 152 bp (Table 1), which corresponds to 96% of the genome size that was estimated by flow cytometry (see above). The sequence of the genome of the ISA1307 strain and the subsequent annotation performed was deposited in the European Nucleotide Archive (ENA, http://www.ebi.ac.uk/ena/data/view/CBTC010000001-CBTC010000154). Although a genome sequence for the type strain *Z. bailii* CLIB213^T^ has been recently published,^15^ this was only released after the assembly of ISA1307 genome. A comparative genomic analysis between the genomes of ISA1307 and *Z. bailii* CLIB213^T^ (discussed below) suggests that the genome of the two parental species are interspersed in the genome of the hybrid strain ISA1307, which shows that the use of *Z. bailii* CLIB213^T^ genome as a reference for the assembly process of ISA1307 genome would have been disadvantageous, compared with the strategy that we have used which was based on the use of *S. cerevisiae* and *Z. rouxii* genomes.

### 3.4. Annotation and structure of ISA1307 genome

To annotate protein-encoding genes in the genome sequence of the ISA1307 strain, a combination of *ab initio* and homology methods were applied using the gene structure of *S. cerevisiae* S288c and *Z. rouxii* CBS732 genes as references. In total, 9925 genes are predicted to be encoded by the genome of the hybrid ISA1307 strain, 90% of these being considered duplicated genes (corresponding to 4385 gene pairs) (Supplementary Table S1) since the encoded proteins share >50% Simap similarity at the amino acid level (listed in Supplementary Table S1). The number of genes predicted to be encoded by the genome of this strain is around twice that of genes annotated for the type strain *Z. bailii* CLIB213^T^.^15^ The scaffolds encoding homologous genes were indicated by suffices ‘A’ and ‘B’ in the scaffold names to reflect the existence of two orthologous sets of scaffold sequences. Sixteen scaffolds lacking clear orthologous sequences remained and were maintained as singletons labelled with the ‘s’ suffix. A MUMmer^44^ alignment of the A and B scaffolds indicates a base identity of 92.6% [for a total of 89.5% (A) and 94.0% (B) of aligned bases], consistent with the proposed hybrid nature of the ISA1307 genome. In line with this difference, variation in the sequence of the ISA1307 duplicated genes was also observed (Supplementary Table S2). For 90% of the ISA1307 duplicated genes, it was found that one of the alleles was almost identical to the corresponding gene found in *Z. bailii* CLIB213^T^ (99–100% identity at the nucleotide level), while the sequence of the other allele was less similar (94–98% identity at the nucleotide level) (Supplementary Table S2). Notably, the ISA1307 gene alleles presumed to have originated from *Z. bailii* (that is, those identical to genes found in the CLIB213^T^ strain) were distributed between A and B scaffolds (Supplementary Table S2), indicating that the genetic information coming from this species is, apparently, not confined to only one of the homeologous chromosomes of the ISA1307 strain probably due to the occurrence of chromosomal rearrangements after hybridization of the parental strains. The differences in the two alleles of ISA1307 duplicated genes registered at the nucleotide level had almost no impact in the sequence of the encoded proteins since only six gene pairs (ZBAI_07571/ ZBAI_01790; ZBAI_06324/ ZBAI_01930; ZBAI_09856/ ZBAI_05001; ZBAI_07267/ ZBAI_01173; ZBAI_08269/ ZBAI_03260; ZBAI_08169/ ZBAI_01798) exhibited a rate of non-synonymous substitutions (dN) and synonymous substitutions (dS) above 1.

The general features of ISA1307 genome, in particular, gene density, average GC content, number of tRNAs and number of rRNA locus are consistent with those described for other hemiascomycetous yeasts, in particular, for *S. cerevisiae* S288c and *Z. rouxii* CBS732^14^ (Table 2). The average gene length of all genes is 1471 bp and the incidence of introns is ∼3%, in line with the results obtained for *S. cerevisiae* S288c and *Z. rouxii* CBS732 (Table 2).^14^ Around 97% (9631 genes) of the genes are predicted to be intron-free. The remaining genes are predicted to have two (277 genes) or three or more exons (17 genes), similarly to the *S. cerevisiae* S288c and *Z. rouxii* CBS732 genes (results not shown). No significant differences were registered in the gene structure located in A and B scaffolds (results not shown), which is compatible with the anticipated genetic relatedness of the parental species that originated the ISA1307 strain.

**Table 2.** General features of ISA1307, *Z. rouxii* CBS732 and *S. cerevisiae* S288c genomes

Strain	No. of chromosomes	Ploidy	Genome size (Mb)	Average GC content (%)	Total no. of CDS	Genome-coding coverage (%)	Average GC in CDS (%)	Average CDS length (bp)	% CDS with introns
ISA1307	13	(∼2*n*)	22	42.4	9931	69.8	43.8	1471	3
*Z. rouxii* CBS138	7	*n*	12.3	39.1	4992	76.1	40.2	1491	3–6
*S. cerevisiae* S288C	16	*n*	12.3	38.3	5769	70.0	40.3	1464	4.5

For the ISA1307 strain genome, each parameter indicated in the table was calculated from the final reconstructed genomic sequence after annotation. ISA1307 genome size was calculated based on the results obtained by flow cytometry shown in Fig. 1A. Average gene density represents the fraction of each genome occupied by protein-coding genes (other genetic elements were not considered). Information from *Z. rouxii* CBS732 and *S. cerevisiae* S288c genomes were taken from^14^ coding sequences (CDS). The size of the chromosomes was estimated based on the results obtained in the PFGE shown in Fig. 1.

**Figure 2.**
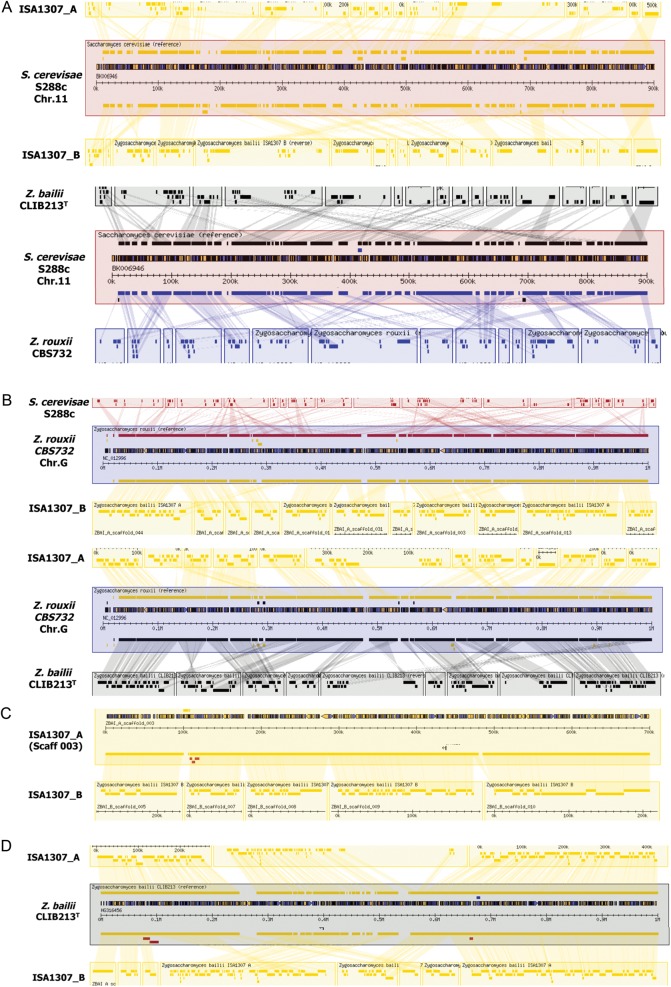
Multigenome alignment of genomic regions of *S. cerevisiae* S288c, *Z. rouxii* CBS732, *Z. bailii* CLIB213T and the interspecies hybrid strain ISA1307. In this picture are shown aligments of *Z. bailii* CLIB213^T^, *S. cerevisiae* S288c, *Z. rouxii* CBS732 and the hybrid strain ISA1307 centered in different genomic regions. Each coloured square represents a different scaffold found in the genomes of *Z. bailii* CLIB213^T^ or of ISA1307 or represents a chromsomes of *S. cerevisiae* S288c or *Z. rouxii* CBS732. Conserved synteny blocks are shown in shaded boxes. This image was obtained using the multigenome alignment GBrowse_syn (http://mips.helmholtz-muenchen.de/gbrowse2/cgi-bin/gbrowse_syn/zbailii).

The sequence and annotation of the genome of the ISA1307 strain disclosed in this study are accessible at http://pedant.helmholtz-muenchen.de/genomes.jsp?Category=fungal, including browsing by a GBrowse instance.^45^ To allow a comparative navigation through the genome of the hybrid strain with the genomes of *Z. rouxii* CBS732, *S. cerevisiae* S288c and *Z. bailii* CLIB213^T^, a GBrowse_syn instance^46^ is accessible under http://mips.helmholtz-muenchen.de/gbrowse2/cgi-bin/gbrowse_syn/zbailii. Massive genomic rearrangements seem to have occurred since the differentiation of *Z. bailii*, *Z. rouxii* and of the ISA1307 strain from *S. cerevisiae* because the genetic information contained in the chromosomes of the budding yeast is dispersed throughout *Z. rouxii* CBS732 chromosomes and throughout the scaffolds of the genome of ISA1307 and of *Z. bailii* CLIB213^T^ (Fig. 2A). The genomes of ISA1307, *Z. bailii* CLIB213^T^ and *Z. rouxii* CBS732 genomes are more syntenic, reflecting the close phylogenetic distance between these strains, however, the existence of large gaps is still evident (Fig. 2B). In general, a high degree of sinteny was observed between the two homeologous scaffolds of the ISA1307 strain and scaffolds of *Z. bailii* CLIB213^T^ (Fig. 2 C and D), consistent with *Z. bailii* being one of the parental species of the ISA1307 strain and with the hypothesis that the other parental strain is phylogenetically close to *Z. bailii*.

Twelve putative centromere-like sequences were found in the 154 scaffolds (Supplementary Table S3) that compose the ISA1307 genome, this being compatible with the 13 chromosomal bands obtained in the PFGE analysis (Fig. 1B). The structure of the centromere sequences obtained is similar to the sequences described for point centromeres of hemiascomycetous yeasts^14^: two conserved domains CDE I and CDE III interspersed by an AT-rich CDE II domain (ranging from 69 to 82%—AT content) (Supplementary Table S3). Although the assembly and annotation above described indicate the existence of large duplicated genomic regions in the genome of the ISA1307 strain, only two or three of the 13 chromosomal bands obtained in the PFGE gel seem to be duplicated (Fig. 1; see above). It is not possible to fully elucidate the structure of ISA1307 genome solely with the data available; however, the results of genome sequencing and karyotyping (Fig. 1) suggest that the genome of this hybrid strain includes chromosomes composed by highly similar homeologous chromosomes (presumably corresponding to the duplicated bands observed in the PFGE gel) and chromosomes composed by more dissimilar homeologous chromosomes (presumably corresponding to the different-sized single bands observed in the PFGE gel). Like ISA1307, other yeast hybrid strains have also been demonstrated to have complex genome structures.^27,29,39,40^

### 3.5 Origin of ISA1307 predicted proteins

The vast majority of the proteins predicted to be encoded by the genome of the ISA1307 strain have their best homologue with proteins found in yeast species phylogenetically close to the *Z. bailii* species, namely *Z. rouxii*, *Torulaspora delbrueckii*, *S. cerevisiae* or other yeasts of the Sacharomycetecea family (results not shown). However, it was possible to identify in the predicted proteome of the ISA1307 strain at least 42 proteins that share a high degree of similarity with proteins found in species distant from the Sacharomycetecea family (e.g. *Candida tenuis*, *Hansenula polymorpha*, *Schizosaccharomyces pombe*) or even in moulds (e.g. *Aspergillus niger*, *Penicillium digitatum* or *Fusarium oxysporum*) (Supplementary Table S4). Six ISA1307 predicted proteins seem to have a bacterial origin since their closest homologues are proteins found in *Burkholderia cenocepacia*, *Burkholderia terrae* or *Dickeya dadantii* (Supplementary Table S4). The occurrence of prokaryote-to-eukaryote and eukaryote-to-eukaryote gene transfers has been demonstrated in *S. cerevisiae* and in several other fungi.^47^ The physiological function of the proteins that seem to have been acquired by the ISA1307 by gene transfer is widespread including a putative Cu, Zn-superoxide dismutase, putative transporters involved in the uptake of monocarboxylates, amino acids and urea, two permeases similar to multi-drug resistance (MDR) transporters of the Major Facilitator Superfamily (MFS), one enzyme required for catabolism of mannose and one enzyme required for metabolization of 1-aminocyclopropane-1-carboxylate, an intermediate in the biosynthesis of the plant hormone ethylene (Supplementary Table S4). Extensive genomic analysis has demonstrated that the acquisition of novel genes by fungi, coming from another fungi or coming from a prokaryote, is often associated with increased cellular fitness to proliferation in the corresponding ecological niche.^47^ Remarkably, 17 of the proteins that seem to have been acquired by the ISA1307 strain do not have an orthologue in *Z. bailii* CLIB213^T^ (Supplementary Table S4), suggesting that they might have been acquired after the hybridization process.

### 3.6. Functional categorization of ISA1307 genes

**Figure 3.**
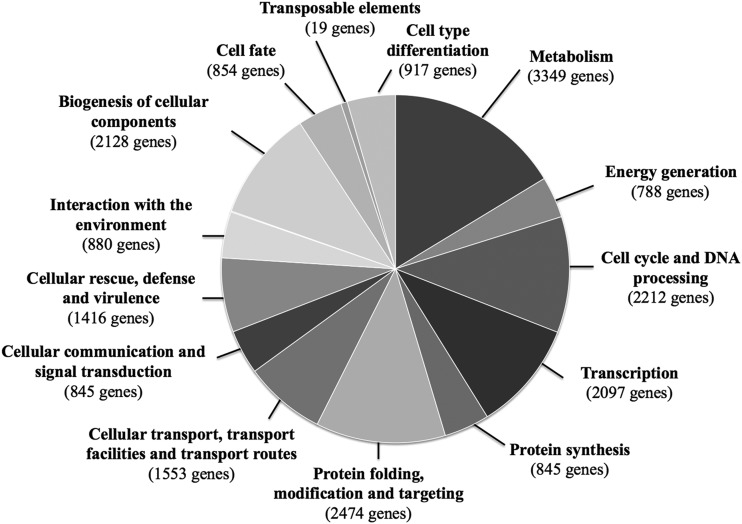
Functional classes of genes predicted to be encoded by the genome of the ISA1307 strain. The genes predicted by the annotation of the genome of the ISA1307 strain (detailed in Section 2) were clustered according to their biological function using the FunCatDB. The number of genes included in each functional category is indicated.

**Figure 4.**
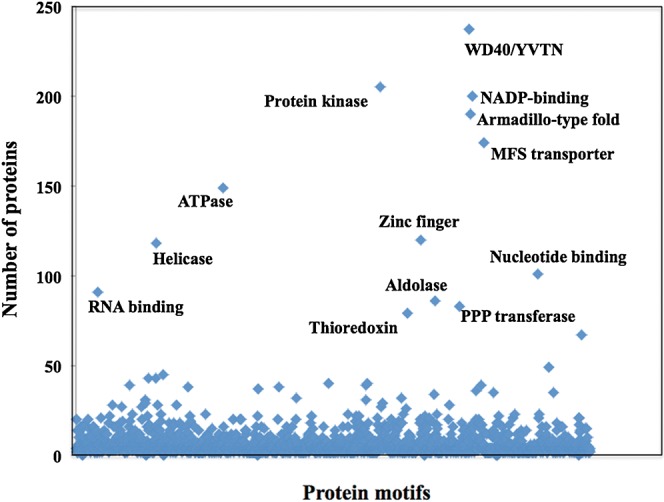
Frequency of putative domains in the ISA1307 predicted proteome. The proteins predicted to be encoded by the ISA1307 genome were searched for putative conserved domains using InterProScan and domains found present in >50 predicted proteins were selected and are highlighted.

The function of the 9931 gene loci predicted to be encoded by the genome of the ISA1307 strain was clustered according to their physiological function using the FunCatDB functional catalogue^48^ (Fig. 3). The highest number of genes were found in the functional classes of ‘Metabolism and generation of energy’ (35% of the total of predicted genes), ‘Protein folding, modification and targeting’ (25% of the predicted genes) and ‘Biogenesis of cellular components’ (21% of the predicted genes) (Fig. 3). The functional categorization of the ISA1307 genome is, in general, similar to the one obtained for *S. cerevisiae* S288c or *Z. rouxii* CBS732 genomes (Supplementary Fig. S1). Genes encoding transposable elements were found to be very scarce in the ISA1307 genome (Supplementary Fig. S1). The more abundant motifs found in the proteins predicted to be encoded by the ISA1307 genome were: (i) the WD40/YTVN motif, present in signal transducing G-proteins or in actin-interacting proteins, (ii) kinases-associated motifs, (iii) motifs present in NADP^+^-binding enzymes, (iv) the armadillo motif, found in protein phosphatases and in initiation translation factors and (v) signature motifs of transporters of the MFS (Fig. 4). These motifs were also the more abundant motifs found in *S. cerevisiae* S288c or *Z. rouxii* CBS732 proteomes (results not shown).

### 3.7. ISA1307 genes involved in metabolism and transport of carbohydrates

Genes encoding enzymes involved in all major pathways of central carbon metabolism were found in ISA1307 ORFeome, including enzymes of the glycolytic pathway, TCA cycle, neoglucogenesis, pentose phosphate pathway and the anaplerotic enzymes isocitrate lyase, pyruvate carboxylase, phosphoenol pyruvate carboxykinase and malic enzyme (Supplementary Fig. S2 and Table S5). Based on the genome annotation, it is anticipated that the respiratory chain of ISA1307 cells includes two mitochondrial NADH dehydrogenases (one located in the inner mitochondrial membrane and another in the outer membrane), one FADH : fumarate dehydrogenase (Complex II), one cytochrome C : ubiquinone reductase (Complex III) and one cytochrome C oxidase (Complex IV) (Supplementary Fig. S2 and Table S5). The hybrid strain ISA1307 does not seem to have a functional complex I, like all the other described yeasts able to perform aerobic alcoholic fermentation,^49^ nor does it have alternative oxidases to perform cyanide-resistant respiration. This organization supports the idea that the hybrid strain ISA1307 obtains energy from the respiratory chain through the proton gradient generated by Complexes III and IV, which is consistent with previous studies demonstrating the high sensitivity exhibited by *Z. bailii* strains to the cytochrome-C reductase inhibitor antimycin.^50^ Genes encoding enzymes required for the catabolism of galactose, glycerol, acetate, ethanol and fructose were also identified in the genome of the ISA1307 strain (Supplementary Fig. S2 and Table S5), consistent with the described ability of this strain to use all these carbon sources.^22^ Enzymes required for catabolism of xylose, sorbose, sorbitol, inulin and glucose-based polysaccharides were also found in the genome of the ISA1307 strain (Supplementary Fig. S2 and Table S5). A putative lactate dehydrogenase (LDH) (encoded by the ZBAI_09900 gene) was also found, suggesting that ISA1307 cells may be able to perform lactic fermentation in alternative to alcoholic fermentation. Interestingly, we could not find in the genome of *Z. bailii* CLIB213^T^, an orthologue for this putative LDH enzyme found in the genome of the ISA1307 strain (Supplementary Table S5), indicating that it may have been acquired from the other parental species of the ISA1307 strain.

Nine putative hexose transporters similar to the well-characterized *S. cerevisiae* Hxt transporters are included in the predicted ‘transportome’ of the ISA1307 strain, as well as two transporters of the sugar porter family, one transporter similar to the *Kluyveromyces lactis* glucose/fructose/galactose transporter Hgt1 and several predicted hexose-like transporters of uncharacterized function (Supplementary Table S5). Fructophilicity, one of the main physiological characteristics that distinguishes the *Z. bailii* species, is retained in the ISA1307 strain.^51^ The activity of the highly specific fructose transporter Ffz1 and the repression of glucose transport by the presence of fructose are considered to be on the basis of fructophilicity of the ISA1307 strain.^5,51^ Besides Ffz1 (ORF ZBAI_03578), three other genes encoding transporters highly similar to Ffz1 were found in the ISA1307 genome (Supplementary Table S5). Three of the four Ffz1-like genes found in the genome of ISA1307 were also present in the genome of *Z. bailii* CLIB213^T^ (Supplementary Table S5). Interestingly, one sugar transporter (ZBAI_01802) that is present in *S. cerevisiae* wine strains but absent in the laboratory strain S288c (Supplementary Table S4) was found in ISA1307 genome. Two gene homologues of *S. cerevisiae* gene *ADY2*, encoding an acetate transporter, and two putative glycerol permeases, were also found to be present in ISA1307 genome (Supplementary Table S4).

### 3.8. Proteins involved in Crabtree effect regulation

The ISA1307 strain and also other strains belonging to *Z. bailii* species are known to have an alleviated Crabtree effect, being able to co-consume glucose and other carbon sources.^6,18,21,22^ The genome sequence of the ISA1307 hybrid strain and of the type strain *Z. bailii* CLIB213^T^ were searched for homologues of the Snf1-signalling pathway, known to play a prominent role in glucose repression in *S. cerevisiae*^52^ (Table 3). No significant differences were registered in the amino acid sequence of the proteins predicted to function in the Snf1-signalling pathway in ISA1307 and in *Z. bailii* CLIB213^T^, indicating that this pathway should function in a similar manner in the two strains (results not shown). However, the organization of the Snf1 pathway in the ISA1307 strain and in *Z. bailii* CLIB213^T^ is apparently different from the one described in *S. cerevisiae*, since the regulatory subunits Gal83 and Sip2 are apparently fused into a single protein (with similarity to the protein domains found in the two independent *S. cerevisiae* proteins) and only two Mig transcription factors are encoded by the genomes of the *Zygosaccharomyces* strains (Table 3). The homology between the three *S. cerevisiae* Mig transcription factors and the two putative Mig-like transcription factors found in *Z. bailii* CLIB213^T^ or in the ISA1307 strain was limited to the DNA-binding domain, suggesting that all these transcription factors may recognize similar DNA-binding sites, as found in other fungi.^53^ Interestingly, the promoter regions of the ISA1307 genes predicted to encode gluconeogenic enzymes, enzymes of the TCA cycle or enzymes required for acetate or glyoxylate metabolism, all subjected to glucose repression in *S. cerevisiae* in a Mig1-dependent manner,^52^ harbour DNA motifs similar to the binding site described for ScMig1 (results not shown). Although a significant difference was registered at the level of the transactivation domains of the two ZbMig and the three ScMig transcription factors, only by the analysis of the genome sequence it is not possible, at this phase, to uncover the mechanisms underlying the different behaviour of the ISA1307 strain and of *Z. bailii* strains, compared with *S. cerevisiae*, concerning the Crabtree effect. The alleviation of the Crabtree effect was suggested to be behind the high intrinsic resistance of the ISA1307 strain, and of the *Z. bailii* species in general, to acetic acid and to other weak acids used as food preservatives.^18,21,22^ However, since these compounds are very diverse in structure, it is unlikely that the high resistance of *Z. bailii* or of the ISA1307 strain to all these weak acids results from the co-catabolism of all these compounds.

**Table 3.** Conservation of the Snf1-signalling pathway in *S. cerevisiae*, in *Z. bailii* CLIB213^T^ and in the interspecies hybrid strain ISA1307

*S. cerevisiae* gene	Function in glucose repression pathway	ISA1307 homologue ORF	*Z. bailii* CLIB213^T^ homologue ORF
*Snf1 pathway*			
*SAK1*	Activates Snf1 kinase by phosphorylation in glucose starvation or non-fermentable carbon sources	ZBAI_08236	BN860_06128g
*SNF1*	Kinase that is activated in response to low glucose concentrations or the presence of non-fermentable carbon; inactivates Mig1 by phosphorylation	ZBAI_02162/ZBAI_08016	BN860_10132g
*SIP1*	Regulatory subunit of Snf1 involved in response to low and high external glucose concentrations	ZBAI_03741	BN860_03840g
*SIP2* *GAL83*	Regulatory subunits of Snf1 that are for activation of the kinase in response to non-fermentable carbon sources	ZBAI_06706/ZBAI_04665	BN860_04170g
*SNF4*	Activating subunit of Snf1; activates glucose-repressed genes and represses glucose-induced genes	ZBAI_01886/ZBAI_06368	BN860_12662g
*MIG1*	Transcriptional repressor of low affinity hexose transporters and of transcription factors Cat8, Hap4 and Adr1 involved in response to non-fermentable carbon sources	ZBAI_06392/ZBAI_06707 ZBAI_00188	BN860_12046g BN860_04148g
*MIG2*	Co-operates with Mig1 in glucose repression		
*MIG3*	Transcriptional regulator required for glucose repression in wild-type *S. cerevisiae* isolates; inactivated in the laboratory strain S288c		

Proteins from ISA1307 and from *Z. bailii* CLIB213^T^ homologous to the *S. cerevisiae* proteins described to belong to the Snf1-signalling pathway.^52^ The physiological function of the *S. cerevisiae* proteins is based on the information available at saccharomyces genome database.

### 3.9. Genes involved in transport and metabolism of amino acids and other nitrogen compounds

The genome of the ISA1307 strain encodes enzymes required for biosynthesis and catabolism of all proteogenic amino acids (Supplementary Fig. S3). Around 40% of the ISA1307 genes included in the ‘Metabolism’ and ‘Cellular transport’ functional classes (Fig. 3) encode proteins related to amino acid metabolism or uptake. Remarkably, there are 18 predicted pyruvate decarboxylases (PDCs) in the genome of the ISA1307 strain while in *Z. rouxii* CBS732 and *S. cerevisiae* S288c there are only three and five proteins, respectively, with this function annotated (Supplementary Table S6). In the type strain *Z. bailii* CLIB213^T^, there are five genes encoding PDC enzymes annotated (Supplementary Table S5), suggesting that the increase in the number of these genes is a particular characteristic of the ISA1307 strain. PDC enzymes are involved in alcoholic fermentation (by catalysing the conversion of pyruvate to acetaldehyde) and in catabolism of branched and aromatic amino acids through the Ehrlich pathway. The amplification of PDC genes in the genome of the ISA1307 strain does not favour alcoholic fermentation since the alcoholic fermentation rate of these cells is below the rates exhibited by *Z. rouxii* or *S. cerevisiae* cells;^7^ however, it may represent an adaptive response to the significant amounts of aromatic and branched amino acids (the main substrates of the Ehrlich pathway) that are found in wines,^54^ the ecological niche where this hybrid strain was isolated from. Thirty-six ISA1307 genes are predicted to encode amino acid permeases, including general amino acid permeases and permeases specific for proline, histidine, lysine, arginine, methionine, histidine, branched amino acids (valine, isoleucine and leucine) and for neutral amino acids (Supplementary Table S6). Genes required for catabolism of allantoine, urea and the non-proteogenic amino acid GABA, as well as genes encoding permeases for these nitrogen sources, were also found in the predicted set of ISA1307 proteins (Supplementary Fig. S3). Interestingly, some of the permease-encoding genes found in the ISA1307 genome have homologues in *S. cerevisiae* strains isolated from wines, but not in the laboratory strain S288c (Supplementary Table S6). The comparison of the genome of several *S. cerevisiae* wine strains with the genome of the laboratory strain S288c strongly suggests that the acquisition of genes required for transport and metabolism of nitrogen sources results from adaptation to the nitrogen-depleted environment of wine musts.^55^

### 3.10. Genes involved in meiosis and mating

Infertility is a common characteristic of hybrid yeast strains due to the incompatibility of genes coming from the parental genomes, gross chromosomal rearrangements and abnormal gene segregation, among other factors.^25,37^ As expected from an hybrid strain, ISA1307 cells were only found to propagate by clonal expansion through mitotic divisions.^17^ Other strains previously classified as *Z. bailii* (NCYC563, NCYC1427, NCYC1416 and NCYC128) were also found to be unable to produce meiotic spores;^36^ however, it remains to be established if these strains do belong to the *Z. bailii* species (not examined in^24^) or if they are hybrid strains or strains belonging to a closely related species. Based on the infertility phenotype exhibited by the ISA1307 strain,^17^ it was generally accepted that *Z. bailii* cells were unable to undergo meiosis. The predicted proteome of the ISA1307 strain and of the type strain *Z. bailii* CLIB213^T^, whose ability to undergo meiosis is, as far as we know, unknown, was searched for proteins homologous to those described to be required for functional meiosis and mating in *S. cerevisiae* (Supplementary Table S7). A number of proteins demonstrated to play an essential role in meiosis in the budding yeast are apparently not encoded by the genome of the ISA1307 strain including: (i) Ime1 and Ume6, key transcriptional regulators of meiosis-related genes, (ii) Emi1, required for transcriptional induction of Ime1, (iii) Zip2 and Cst9, involved in the formation of the synaptonemal complex, (iv) Don1, Mpc54, Spo16, Spo20, Spo21, Spo22 and Spo74, involved in the formation of the meiotic plate and (v) Rec104, Zip2, Mlh2, Msh4 and Msh5 genes, required for the induction of meiotic recombination (Supplementary Table S7). Concerning the molecular machinery required for mating in *S. cerevisiae*, the transcription factors Dig1 and Dig2, required for the regulation of mating-specific genes and for triggering the invasive growth pathway, also seem to be absent from the ISA1307 genome (Supplementary Table S6). Moreover, neither the *S. cerevisiae a* or α matting cassettes (encoded in the *HMRA1*/*HMRA2* and *HMALPHA1*/*HMALPHA2* locus) nor the corresponding *a* or α mating factors [encoded by the MATa1/MATa2 and MAT(alpha1)/MAT(alpha2) genes] were also found to have homologues in the ISA1307 genome (Supplementary Table S6). Around half of the genes required for meiosis in the budding yeast that are missing in the genome of the ISA1307 strain were found in the genome sequence available for *Z. bailii* CLIB213^T^; however, this strain lacks proteins with a very prominent role in the meiotic process, such as Ime1 (Supplementary Table S7). Homologues to the *S. cerevisiae a* and α mating cassettes were also not found in the genome of *Z. bailii* CLIB213^T^ (Supplementary Table S7). Solely based on the inspection of the genome sequence, it is not possible to say if the infertility phenotype of ISA1307 cells derives from being a hybrid strain or if this trait was found in the parental species, in particular, in *Z. bailii*.

### 3.11. ISA1307 genes involved in stress response

One of the goals underlying species hybridization is the increase in cell robustness. Indeed, hybrid yeast strains isolated from the harsh environmental conditions of wine fermentations were found to be more resistant to stress than their parental species.^56–58^ One of the main phenotypic traits of ISA1307 strain is its high tolerance to acetic acid stress.^18,21^ *Zygosaccharomyces bailii* species are known for being resistant to stress induced by several weak acids food preservatives and tolerant to several sanitizers and to osmotic stress induced by high sugar concentrations.^2^ Although this high intrinsic resilience of *Z. bailii* cells to the above referred stresses is believed to underlie the high spoilage capacity of this yeast species,^2^ specially in acidic foods and drinks, the molecular mechanisms behind this trait are still unknown or unclear. Having this in mind, the genome of the ISA1307 strain was searched for proteins described to play a role in in *S. cerevisiae* response and resistance to weak acid food preservatives, in particular, to acetic acid. The genome of the ISA1307 strain encodes one protein, encoded by the paralogous genes ZBAI_03527 and ZBAI_08525, homologous to the *S. cerevisiae* transcription factors Msn2 and Msn4, which control the transcriptional response to environmental stress,^59^ in particular the response to weak acid food preservatives.^13^ Apparently, *Z. bailii* CLIB213^T^ genome also encodes one protein (ZYBA0S17-00848g1_1) with similarity to the ScMsn2/ScMsn4 transcription factors. The highest degree of similarity of this putative ZbMsn2/4 with ScMsn2 or ScMsn4 is registered at the level of the DNA-binding domain, mapped in the C-terminal region of these proteins.^59^ Most of the genes involved in *S. cerevisiae* Environmental Stress Response (ESR) are conserved in the genome of the ISA1307 strain and, in general, the promoter region of these putative stress-responsive genes harbours the STRE motif (5′-CCCCT-3′, results not shown) for ScMsn2/ScMsn4 binding. Approximately 98% of genes that were found to mediate MDR in *S. cerevisiae*^60^ are conserved in ISA1307 and *Z. bailii* CLIB213^T^ genomes (Supplementary Table S8), suggesting that some of the mechanisms that were described to underlie the MDR phenomenon in the budding yeast, namely plasma membrane lipid composition, intracellular protein trafficking mediated by vesicular transport or proteosomal activity, may also be active in *Z. bailii*. The genome of the ISA1307 strain encodes at least 63 MDR transporters of the ABC (28) and of the MFS (35) (Supplementary Table S9). The role of a number of these transporters in *S. cerevisiae* MDR has been well documented, in particular, the MFS transporters Azr1, Aqr1, Tpo2 and Tpo3,^61^ and the ABC transporter Pdr12,^62^ described as determinants of *S. cerevisiae* resistance to weak acids food preservatives^13^ (Supplementary Table S9). Four non-paralogous ISA1307 genes are predicted to encode Pdr12-like proteins; this apparent *PDR12* amplification being an interesting observation considering the major role attributed to this protein in *S. cerevisiae* response and resistance to weak acid-induced stress.^13^ Six MFS-MDR transporters of uncharacterized function that do not appear to have homologues either in the sequenced *S. cerevisiae* strains or in *Z. rouxii* CBS732 were also found to be encoded by the genome of the ISA1307 strain (Supplementary Table S9). Two of these transporters (encoded by ZBAI_07578 and by the paralogous genes ZBAI_00386/ ZBAI_01804) do have a very high homology to MFS-MDR transporters from *Candida dubliensis* and *Aspergillus fumigatus*, suggesting that these genes could have been acquired by gene transfer (Supplementary Table S9). Interestingly, we could not identify in the genome of *Z. bailii* CLIB213^T^ orthologues for these putative eight MFS-MDR transporters nor for the four Pdr12-like genes that were found in the genome of the ISA1307 strain (Supplementary Table S9). Therefore, it is hypothesized that these genes could be encoded by the non-*Z. bailii* species genome of the hybrid strain ISA1307. The vast majority of the genes that mediate *S. cerevisiae* tolerance to acetic acid, propionic acid and sorbic acids (90–95%, depending on the weak acid) were also found to be conserved in the ISA1307 hybrid strain and in *Z. bailii* CLIB213^T^ genomes (Supplementary Table S10). Among these, conserved genes are the key regulators of *S. cerevisiae* response to weak acid stress Haa1, War1 and Rim101.^13^ Most of the genes of the Haa1-, Rim101- or War1- regulons that were described in *S. cerevisiae* were also found in the ISA1307 predicted proteome (Supplementary Table S10), suggesting that these signalling pathways could also be active and play a role in the intrinsic high resistance of this strain and of the *Z. bailii* species to weak acids food preservatives and, in particular, to acetic acid. Although the stress signalling pathways described for *S. cerevisiae* are well conserved in other fungi, there is evidence for a rapid adaptive evolution of these regulatory pathways under the environment challenges to which they are exposed in the different ecological niches.^63^ The knowledge of the genome sequence of the ISA1307 interspecies hybrid strain opens the door to the *in silico* and *in vivo* genome-wide identification of genes and pathways involved in stress resistance in *Z. bailii* and in this *Z. bailii*-derived hybrid strain, in particular, of those genes relevant for yeast protection against stresses characteristic of the wine environment.

## Supplementary data

Supplementary Data are available at www.dnaresearch.oxfordjournals.org.

## Funding

This research was supported by FCT and FEDER through POFC-COMPETE [contracts PEst-OE/EQB/LA0023/2011_research line: Systems and Synthetic Biology PTDC/AGR-ALI/102608/2008, PEst-C/BIA/UI4050/2011, and post-doctoral grant to M.P. (SFRH/BPD/73306/2010) and PhD grants to J.F.G. (SFRH/BD/80065/2011) and F.C.R. (SFRH/BD/82226/2011)]. U.G. acknowledges the Austrian Science Fund (FWF, special research project F3705).

## Supplementary Material

Supplementary Data
