# Supplementary material for: The Genome Sequence of the Highly Acetic Acid-Tolerant Zygosaccharomyces bailii-Derived Interspecies Hybrid Strain ISA1307, Isolated From a Sparkling Wine Plant
Source: DNA Res. 2014 Jan 21;21(3):299–313. doi: 10.1093/dnares/dst058 (PMC4060950; doi:10.1093/dnares/dst058)
Supplement: Supplementary Data [file supp_21_3_299__index.html]

The Genome Sequence of the Highly Acetic Acid-Tolerant Zygosaccharomyces bailii-Derived Interspecies Hybrid Strain ISA1307, Isolated From a Sparkling Wine Plant — The Genome Sequence of the Highly Acetic Acid-Tolerant Zygosaccharomyces bailii-Derived Interspecies Hybrid Strain ISA1307, Isolated From a Sparkling Wine Plant — Supplementary Data 

# The Genome Sequence of the Highly Acetic Acid-Tolerant *Zygosaccharomyces bailii-*Derived Interspecies Hybrid Strain ISA1307, Isolated From a Sparkling Wine Plant

## Supplementary Data

Supplementary Data

**Files in this Data Supplement:**

- Supplementary Data - Pdf file
- Supplementary Data - Supplementary Data
- Supplementary Figure 1 - pdf file
- Supplementary Figure 2 - tif file
- Supplementary Figure 3 - tif file
- Supplementary Table 1 - xlsx file
- Supplementary Table 2 - xlsx file
- Supplementary Table 3 - xlsx file
- Supplementary Table 4 - xlsx file
- Supplementary Table 5 - xlsx file
- Supplementary Table 6 - xlsx file
- Supplementary Table 7 - xlsx file
- Supplementary Table 8 - xlsx file
- Supplementary Table 9 - xlsx file
- Supplementary Table 10 - xlsx file
