# Supplementary material for: The Genome Sequence of the Highly Acetic Acid-Tolerant Zygosaccharomyces bailii-Derived Interspecies Hybrid Strain ISA1307, Isolated From a Sparkling Wine Plant
Source: DNA Res. 2014 Jan 21;21(3):299–313. doi: 10.1093/dnares/dst058 (PMC4060950; doi:10.1093/dnares/dst058)
Supplement: Supplementary Data [file supp_dst058_dst058supp.pdf]

Methods used for sequencing, assembly and annotation of the genome of the *Z. bailii* derived interspecies hybrid strain ISA1307.

Sequencing

Genomic DNA from ISA1307 strain was sheared by nebulization to generate DNA fragments that were used for construction of the DNA library. DNA libraries (20 ng/µl) were constructed by ligating the specific oligonucleotides (Illumina adapters with around 80 mers in size) designed for PE sequencing to both ends of DNA fragments with the TA cloning method. The ligated DNA was then size selected on a 2% agarose gel. DNA fragments of ~ 500 bp were excised from the preparative portion of the gel. Taking into account the size of the adapters the insert size of the library is in the range of 250-350 bp. DNA was finally recovered using a Qiagen gel extraction kit and was PCR amplified to produce the final DNA library. Five picomoles of DNA from each strain were loaded onto two lanes of the sequencing chip, and the clusters were generated on the cluster generation station of the GAIIx using the Illumina cluster generation kit. Bacteriophage X174 DNA was used as a control. In the case of paired-end reads, distinct adaptors from Illumina were ligated to each end with PCR primers that allowed reading of each end as separate runs. The sequencing reaction was run for 100 cycles (tagging, imaging and cleavage of one terminal base at a time), and four images of each tile on the chip were taken in different wavelengths for exciting each base-specific fluorophore. For paired-end reads, data were collected as two sets of matched 100-bp reads. Reads for each of the indexed samples were then separated using a custom Perl script. Image analysis and base calling were done using the Illumina GA Pipeline software.

Assembly and annotation

Short reads were assembled using SOAPdenovo (http://soap.genomics.org.cn)[1], a genome assembler developed specifically for use with next-generation short-read sequences. As the algorithm is sensitive to sequencing errors, the reads were filtered for low quality reads using the DynamicTrim and LengthSort Perl scripts within SolexaQA and only high-quality reads were used for the de novo assembly. These scripts trimmed each read to the longest contiguous read segment for which the quality score at each base was greater than p = 0.05 (approximately equivalent to a Phred score of 13), and then removed sequence reads shorter than 25 bp respectively. When one sequence of a pair was removed, the remaining sequence was put into a separate file and used as a singleton during de novo assembly. SOAP GapCloser was used to close gaps whenever possible. The obtained scaffolds were sequentially ordered based on their level of synteny with the genomes of *Z. rouxii* CBS732 and *S. cerevisiae* S288c. To confirm correct scaffold positioning, specific primers hybridizing at the end or at the beginning of contiguous scaffolds were designed and the amplified PCR product was sequenced to confirm the scaffold order and

eventually to close gaps. To predict genes in the genome of the ISA1307 strain two ab initio gene predictor algorithms were used, GeneMark-S and GenMark-ES version 2.3[28,29]. Gene models differently predicted by the algorithms were manually curated based on the structure obtained for *Z. rouxii* CBS 732 and *S. cerevisiae* S288c homologues. The genomes were analyzed using the Pedant system[37] to allow comparative feature analysis which includes computation of the Similarity Matrix of Proteins (SIMAP)[30]. The SIMAP database provides a comprehensive calculation of protein sequence similarities/identities, sequence-based features and protein function predictions. Amino acid identities of homologous stretches are multiplied by the length of the homologous region and divided by the length of the whole protein resulting in the 'Simap similarity'.

[1] Li, R., H. Zhu, J. Ruan, W. Qian, X. Fang, Z. Shi, Y. Li, S. Li, G. Shan, K. Kristiansen, H. Yang, and J. Wang, De novo assembly of human genomes with massively parallel short read sequencing. Genome Res, 2010. 20(2): p. 265-72.
