# Supplementary material for: The Genome Sequence of the Highly Acetic Acid-Tolerant Zygosaccharomyces bailii-Derived Interspecies Hybrid Strain ISA1307, Isolated From a Sparkling Wine Plant
Source: DNA Res. 2014 Jan 21;21(3):299–313. doi: 10.1093/dnares/dst058 (PMC4060950; doi:10.1093/dnares/dst058)
Supplement: Supplementary Data [file supp_dst058_dst058supp1.pdf]

Molecular typing of the ISA1307 strain

Taxonomic classification of the ISA1307 strain was performed based on the comparison of nucleotide sequence of *RPB1*, *RPB2*, *EF1-a* and β-tubulin-encoding gene) with DNA sequences deposited at GeneBank. The sequence of *RPB1*, *RPB2,* β-tubulin and *EF1-α* genes was obtained from the genome sequence. The table shows the results obtained with the 3-top hits obtained for each gene sequence as well as the nucleotide variations (including gaps) observed. The two gene copies of *RPB2*, EF1-α and β-tubulin found in ISA1307 genome were named 1 and 2.

Gene of the ISA1307 strain	Top similar hits	Nucleotide variations
RPB1_1 (ZBAI_00500)	1. *Z. bailii* CLIB 213^{T} (=ATCC 58445)	0 (660/660)
	2. *Z. bailii* ATCC 8766	0 (660/660)
	3. *Z. bailii* ATCC 38924	0 (660/660)
RPB2_1 (ZBAI_09456)	1. *Z. bailii* CLIB 213^{T} (=ATCC 58445)	1 (1031/1032)
	2. *Z. bailii* ATCC 38924	1 (1032/1033)
	3. *Z. bailii* ATCC 8766	2 (1031/1033)
RPB2_2 (ZBAI_03774)	1. *Z. parabailii* ATCC 60483	43 (990/1032)[*]
	2. *Z. parabailii* ATCC 8099	44 (989/1032)[*]
	3. *Z. parabailii* ATCC 56075	45 (988/1032)[*]
EF1-α_1 (ZBAI_05667)	1. *Z. bailii* CLIB 213^{T} (=ATCC 58445)	0 (932/932)
	2. *Z. pseudobailii* ATCC 56074	23 (909/932)
	3. *Z. parabailii* ATCC 56075	19 (913/932)
EF1- α_2 (ZBAI_00412)	1. *Z. parabailii* ATCC 56074	1 (871/872)
	2. *Z. bailii NRRL Y-2228*	16[**] (853/869)
	3. *Z. bailii* CLIB 213^{T} (ATCC58445)	18 (854/872)
β-tubulin_1 (ZBAI_01412)	1. *Z. parabailii* ATCC 56075	36 (846/882)
	2. *Z. bailii* CLIB 213^{T} (=ATCC 58445)	36 (846/882)
	3. *Z. pseudobailii* ATCC 56074	57 (825/882)
β-tubulin_2 (scaffold 17:12075-12296)	1. *Z. bailii* CLIB213^{T} (=ATCC 58445)	1 (881/882)
	2. *Z. parabailii* ATCC 56075	37 (845/882)
	3. *Z. pseudobailii* ATCC 56074	41 (841/882)

[*] The differences identified correspond to ambiguous positions (W, R or Y) described in *Z. pseudobaili* and *Z. parabailii* strains; [**] The different nucleotides correspond to 'Ns' in strain Y-2228; [**]
