# Supplementary figures and images for: The Genome Sequence of the Highly Acetic Acid-Tolerant Zygosaccharomyces bailii-Derived Interspecies Hybrid Strain ISA1307, Isolated From a Sparkling Wine Plant
Source: DNA Res. 2014 Jan 21;21(3):299–313. doi: 10.1093/dnares/dst058 (PMC4060950; doi:10.1093/dnares/dst058)

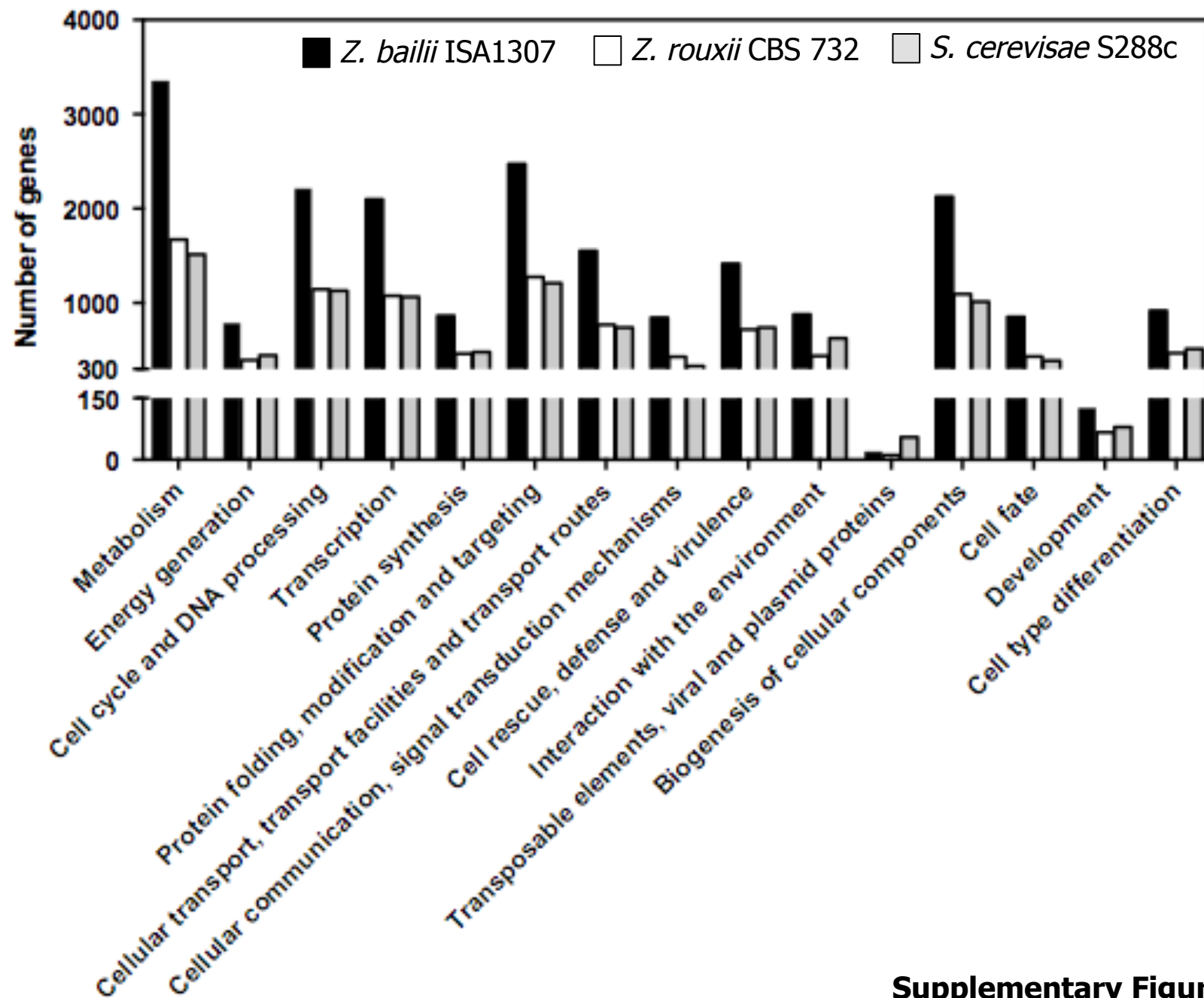

**Supplementary Figure S1**

Supplement: Supplementary Data [file supp_dst058_dst058supp_fig1.pdf]

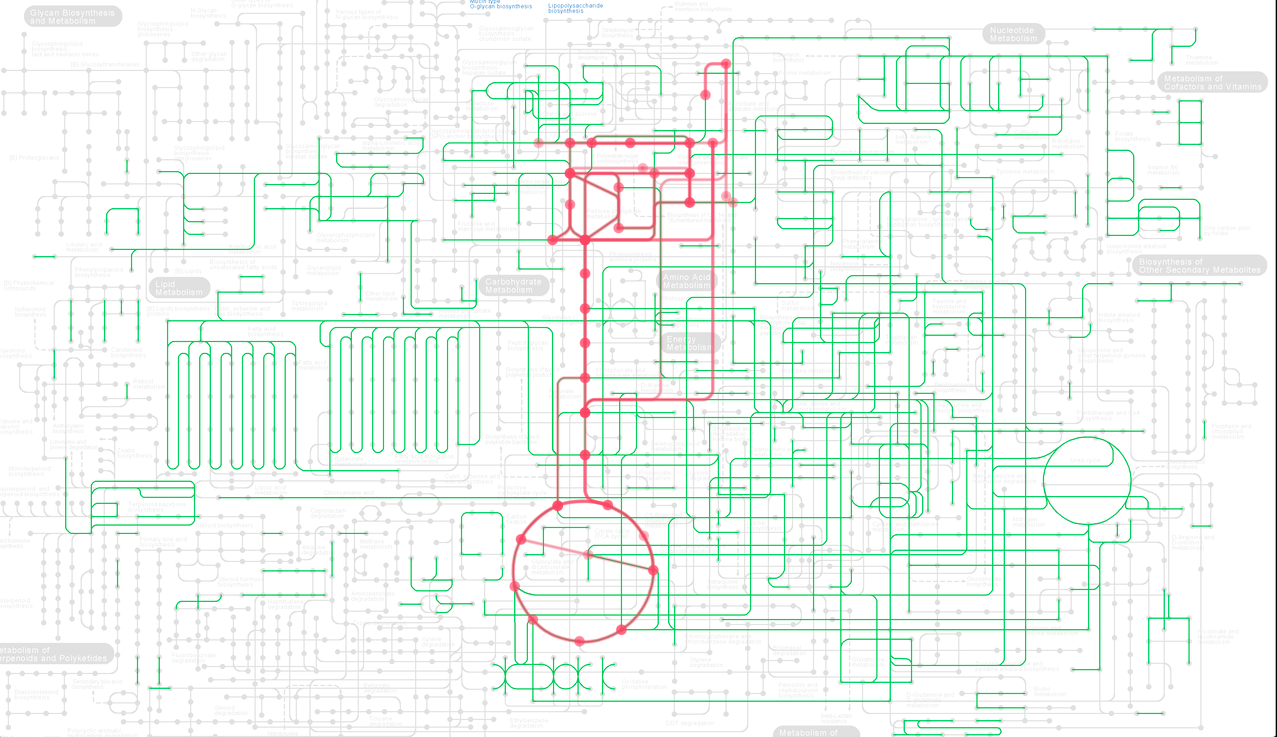

Supplement: Supplementary Data [file supp_dst058_dst058supp_fig2.tif]

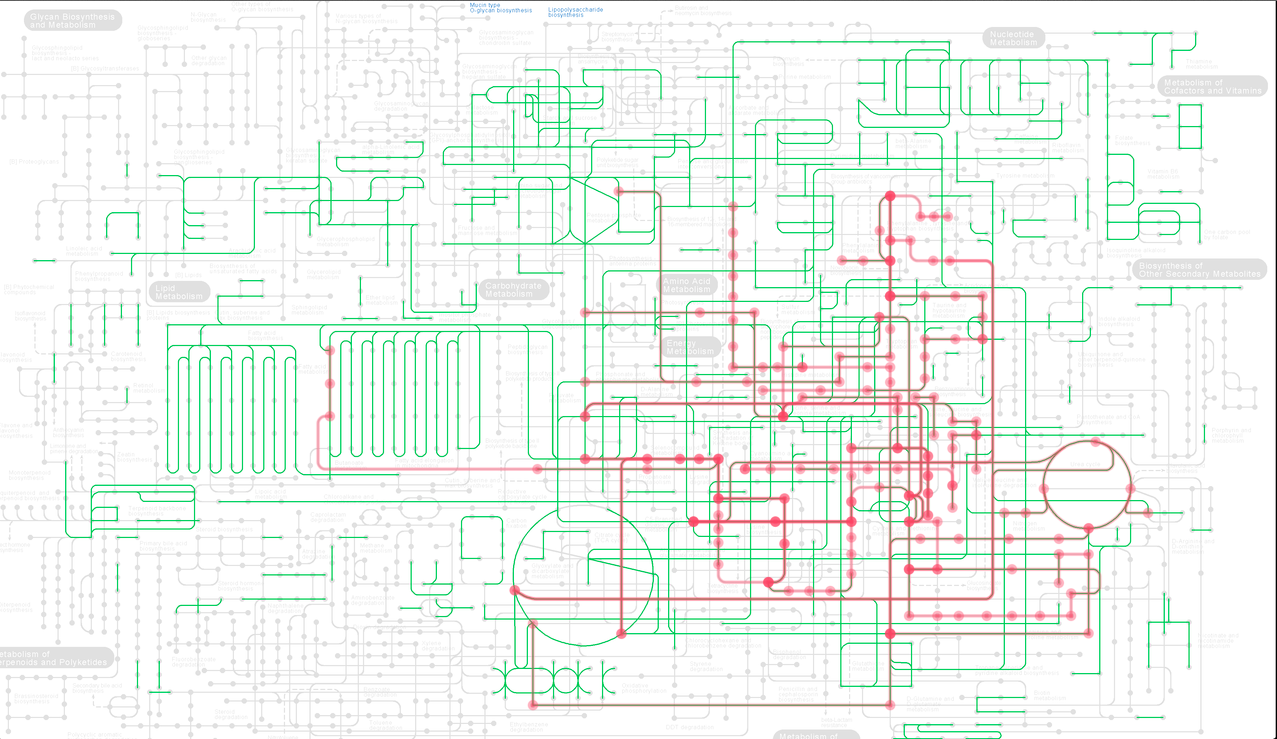

Supplement: Supplementary Data [file supp_dst058_dst058supp_fig3.tif]
